# Psychotherapeutic Treatment of Attachment Trauma in Musicians with Severe Music Performance Anxiety

**DOI:** 10.3390/bs15091270

**Published:** 2025-09-17

**Authors:** Dianna Kenny

**Affiliations:** University of Sydney, Sydney 2006, Australia; dianna.kenny@sydney.edu.au

**Keywords:** severe music performance anxiety, attachment theory, attachment-informed psychotherapy, intensive short-term dynamic psychotherapy, life history

## Abstract

The aim of this paper is to contribute to the further development of a coherent theory of music performance anxiety (MPA) and its treatment. I have previously proposed three forms of MPA-focal, MPA with social anxiety, and MPA with panic and/or depression. An attachment disorder was proposed as a possible underlying psychopathology for this third type of MPA. Accordingly, open-ended in-depth assessment interviews of three professional musicians presenting with severe MPA that included panic attacks and depressed mood were analyzed from an attachment theory perspective. Two of these musicians participated in short-term psychodynamic psychotherapy. It was hypothesized that the musical performance setting re-triggers unprocessed feelings related to early attachment trauma, and that performance anxiety can be a manifestation of the emergence into consciousness of these powerful early feelings. As hypothesized, severely anxious musicians suffered both early and current relational trauma that was expressed through symptomatology in their MPA manifestations. The assessment interview of the first musician demonstrated how MPA can arise in the midst of other challenging current life circumstances that re-trigger feelings about early attachment failures and the importance of taking a full life history from severely performance-anxious musicians. Excerpts from the two musicians’ short-term psychodynamic psychotherapy demonstrated resolution of their severe MPA. Failure to identify and treat underlying attachment disorders in severely anxious musicians may render other forms of treatment ineffective or short-lived.

## 1. Introduction

Until recently, MPA (MPA) has been understood as a unidimensional construct occurring on a continuum of severity from career stress at the low end to stage fright at the high end (Brodsky, 1996). The clinical treatment and study of musicians suffering severe MPA (Kenny, 2004, 2005a, 2005b, 2009, 2011, 2016; Kenny & Holmes, 2015, 2018) alerted the author to the complex underlying psychopathology that appears to be universally present in this subgroup. Addressing only the “surface” symptomatology, such as the worrisome physiological arousal (sweaty palms; tremors, trembling; dry mouth, gastrointestinal upset) and the cognitive distortions (chronic worry, rumination, catastrophizing, etc.), has not been shown to effect a worthwhile or lasting change in performance distress experienced by these musicians.

Kenny (2011) argued that MPA is better understood as a typology comprising three subtypes to account for qualitative differences in presentation as well as variations in severity. The three subtypes are as follows: (i) MPA as a focal anxiety, where there is no generalized social anxiety, depression, or panic; (ii) MPA with other social anxieties; and (iii) MPA with panic, generalized anxiety, and depression. There are different levels of severity within each subtype. The theoretical model underpinning this typology is that MPA represents an intersection between an individual’s developmental history, which may be more or less disturbed—mildly, or not at all, in the case of focal anxiety and more severely in the third subtype—and the specific motor, cognitive, and psychosocial challenges of musicianship, talent, achievement of technical mastery, preparedness, performance demands, exposure, competitiveness, and so on. Accordingly, MPA will have some of the general characteristics of other psychological disorders, in particular, anxiety disorders, which are shared with non-musicians, and some that are specific to MPA and other performing artists, such as dancers, actors, and athletes. This conceptualization of performance anxiety awaits further empirical exploration. The aim of this paper is to contribute to a better representation and understanding of the third proposed subtype of MPA and to stimulate further research of this subgroup of anxious musicians using two related theoretical frameworks, attachment-informed psychotherapy and intensive short-term dynamic psychotherapy, which has evolved from attachment-informed psychotherapy.

### 1.1. Attachment Theory

Attachment theory (Ainsworth, 1963; Bowlby, 1973) offers a heuristic, evidence-based framework from which to explore severe performance anxiety in professional musicians. Attachment is defined as a biologically based motivational–behavioral system whose primary goal is to ensure the survival of the helpless infant. This system is characterized by three features: (i) maintenance of the infant’s physical proximity to its caregiver through crying, clinging, crawling, searching and reaching for the attachment figure to attain physical closeness; (ii) using the attachment figure as a “secure base” (Ainsworth, 1963) from which to explore the environment; and (iii) returning to the primary attachment figure (usually the mother) as a “safe haven” when in danger or alarmed (Kenny, 2011). Bowlby (Bowlby, 1988) later expanded his view of the role of attachment to include reassurance of the ongoing (emotional) availability of the caregiver, experienced as “felt security” (defined as a subjective or internal experience of comfort and safety (Holmes, 2010; Sroufe & Waters, 1977), recently redefined as “epistemic trust” (Fonagy, 2015)).

Four main patterns of attachment have been identified (Ainsworth, 1963; Main, 1995). First, securely attached infants, with attuned and responsive caregivers, develop felt security. Insecure infant–parent dyads fall into three types: (i) in avoidant or “deactivating” attachment (Mikulincer & Shaver, 2011); (ii) ambivalent or “hyper-activating” attachment; and (iii) disorganized attachment. Avoidant infants appear calm and independent and more interested in exploring the environment than communicating with their caregiver. Despite the apparent lack of manifest distress, these infants have elevated heart rates and levels of circulating cortisol (stress hormone) (Letourneau et al., 2011). These infants learn that attempts to seek comfort and care from their mothers are likely to be met with rebuff and therefore develop self-sufficiency. In ambivalent or “hyper-activating” attachment, infants exposed to inconsistent and intermittent care are too concerned about their mothers’ whereabouts to feel free to explore their environment. They respond with intense distress when left, and appear inconsolable when reunited (Mikulincer & Shaver, 2011). In disorganized attachment, that presages later psychopathology, infants have experienced “frightened and/or frightening” caregivers (Lyons-Ruth et al., 2015) and are thus subject to an “approach-avoidance dilemma” in which the source of comfort is also a potential threat, either through neglect or active physical or sexual harm from parents who are mentally ill, substance affected, or chronically depressed or anxious (Main et al., 2005). Following reunion after separation, disorganized infants may manifest this conflict through seemingly inexplicable and bizarre behaviors, which may have a “perverse” self-soothing component, that includes “freezing”, collapsing to the floor, and appearing dazed and confused.

The development of these varying attachment patterns—which also have a severity as well as a categorical dimension (Maunder et al., 2006)—is an interpersonal process that emerges in the context of the parents’ caregiving style. Parental states of mind are typically assessed using the Adult Attachment Interview (AAI) (Hesse, 2008). Secure mothers tend to have secure infants who grow to be secure adults. Parents of avoidant infants tend towards dismissing states of mind; they minimize or devalue the influence of their own attachment experiences, and ignore or suppress their infants’ attachment needs, who in turn, learn to minimize their own needs, becoming compulsively self-reliant and reluctant to feel or express emotions. Such parents, however, tend to display excessive physiological arousal, as do their avoidant infants (Spangler & Grossmann, 1993). Parents of ambivalent infants have “preoccupied” states of mind (Main et al., 2005), so called because past unsatisfactory attachment experiences intrude upon their present life and relationships. The emotional life of such parents is governed by feelings of helplessness and fears of abandonment, disapproval, or rejection; hence, they are often discouraging of their child’s growing autonomy. In contrast to avoidant infants, ambivalent infants use hyper-activating strategies that amplify their affect in an attempt to secure the attention of their unreliably available parents (Cassidy & Berlin, 1994). Finally, parents of disorganized/unresolved infants have often suffered repeated trauma in their own developmental histories and are classified as “unresolved” on the AAI. Responses to unresolved trauma include fear, emotional withdrawal, and dissociation (Madigan et al., 2006).

Although the attachment patterns of anxious musicians have received little attention, several studies have pointed to the importance of musicians’ experiences of their parents, and how those experiences shape the life schemas or cognitive templates and psychopathology that underpin a person’s mode of operating in the world (Kantor-Martynuska & Kenny, 2018; Kenny & Holmes, 2015, 2018). Empirical support for the detrimental impact of overcontrolling, overinvolved parenting in young musicians’ autonomy, feelings of competence, intrinsic motivation, engagement in music, and general self-efficacy suggests that parenting experiences in childhood may have an important role in mitigating or exacerbating the development of MPA (Kirsner et al., 2023). An earlier study (Wiedemann et al., 2020) reported a moderate association between MPA and parenting style. Of interest to this discussion, however, is that attachment behavior classification discriminated higher from lower MPA subgroups. Musicians with a positive self-concept (dismissive or secure attachment) displayed lower MPA; musicians with a negative self-concept (preoccupied or anxious attachment) displayed higher MPA. The effect of parenting experiences and attachment style on MPA was related to the presence of generalized anxiety. This study used only 24 items from the K-MPAI that the authors believed were related to performance. This unusual selection of items precludes direct comparisons with other studies examining the links between MPA, parenting style, attachment style, and other psychopathologies. Schoonover (2025) has developed a number of possible interventions based on this research to support anxious musicians, including developing more caring music learning environments whose teachers resemble the behavior of attuned parents.

How might we understand these relationships? A number of brain structures are involved in regulating the brain’s danger response system, including the amygdala, mature at birth, which is involved in the fight/flight response, and where, it is hypothesized, unconscious emotional memories are encoded; and the later maturing hippocampus, which moderates the reactions of the amygdala and interacts with the cortex to store explicit, linguistically retrievable memories (Le Doux, 1996). In children who have suffered severe emotional or relational trauma, the development of these brain structures may be compromised, with the result that the unchecked reactivity of the amygdala will produce extremely intense autonomic reactions in response to relatively minor internal or external triggers (Wallin, 2007). The extreme reactions of intense MPA in some musicians can perhaps be understood in this context. Kenny (2011) has hypothesized that severely performance-anxious individuals, by virtue of faulty attachment experiences in early life, do not develop a sense of felt security on which to draw when endangered on stage.

The attachment system remains active during adulthood and continues to exert a significant influence on psychological and social functioning. Adults respond to perceived threats with activation of the mental representations of attachment figures laid down in infancy and childhood, as a means of coping and regulating emotions (Ein-Dor et al., 2010). When these attachment systems are insecure, especially if disorganized, their activation at times of stress and crisis is likely to result in emotional dysregulation. This provides a plausible model for the type of emotional difficulties experienced by musicians whose MPA feels unmanageable. Individuals with developmental histories leading to patterns of severe insecure attachment cannot readily mitigate distress or attain felt security. Instead, in the face of intense distress, alternative, secondary attachment strategies involving either hyper-activation or deactivation of the attachment system are triggered (Main et al., 2005). By contrast, those who are securely attached demonstrate both a strong sense that they can manage the threat and, if need be, seek support from others to aid their own coping efforts (Shaver et al., 2009).

### 1.2. Attachment-Informed Psychotherapy (AIP)

**Figure 1:**
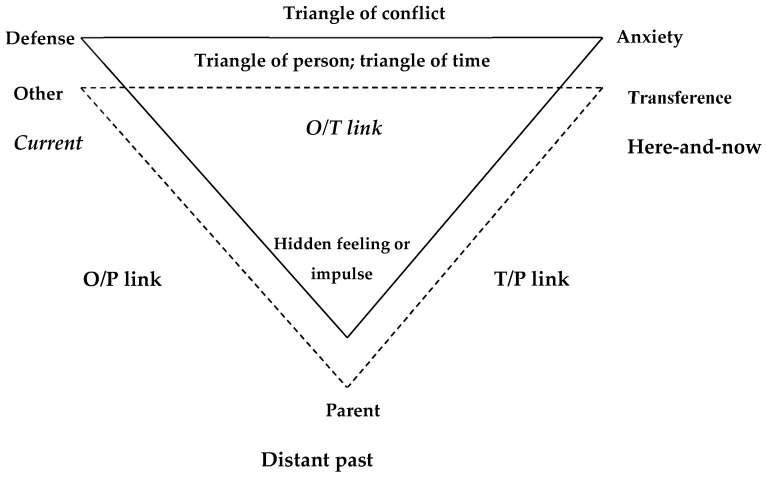
Triangles of conflict, person, and time and the links between them that are made in psychotherapy (Ezriel, 1952; Malan, 1979).

Attachment-informed psychotherapy (AIP) is a short-term psychotherapy that shares with other short-term psychotherapies some common features, which include maintaining a therapeutic focus (as opposed to the free association of psychoanalysis), active therapist involvement (as opposed to the non-intrusiveness of psychoanalysts), and the explicit use of the transference. AIP uses the triangle of conflict (feelings, anxiety, and defense; Ezriel, 1952) and the triangle of person/time [past (parents), therapist, and current relationships; Menninger (1958) to maintain the therapeutic focus (Grieco, 2005). Figure 1 presents a schematic representation of how the triangles guide the therapeutic process. Keeping these triangles in mind, the therapist interprets the patient’s reactions and behavior according to the linkages in his/her triangle. Three linkages are interpretable for each triangle—these are the other (current relationship)–therapist link (O/T); therapist–parent link (T/P), and other/parent (O/P). For a detailed explanation, see Kenny (2011). This method of case formulation has been widely adopted in psychodynamic circles (Hoffman, 2019; Julien & O’Connor, 2017; Kenny, 2016; Kenny & Holmes, 2015, 2018; Leichsenring & Salzer, 2014; Molnos, 1984).

Particular attention is directed to the role of anxiety, which is viewed either as a response to an external threat or an internal, emotional conflict. In situations where a legitimate external threat exists, anxiety is an adaptive response that prepares the individual to deal with the threat as effectively as possible. Internal emotional conflicts are created through ruptures in attachment relationships in the first eight years of life (Pauli-Pott & Mertesacker, 2009). There is a wide range of events and situations that create attachment ruptures. These include, but are not limited to death of a parent, prolonged separation due to the illness of a child or parent, emotional neglect, emotional, physical, or sexual abuse, or a more subtle but equally damaging chronic parental misattunement to, or lack of empathy with, their children’s emotional signals. The age of the child at the time the rupture first occurs, and the frequency and duration of these experiences of rupture are indicative of the severity of the attachment rupture (Bond, 2010). The younger the child, the more frequently the events occur, and the longer the overall duration of the events, or the more persistent and unrelieved the parental misattunement, the more severe is the attachment rupture (Beebe et al., 2010; Bowlby, 1960, 1973).

### 1.3. Intensive Short-Term Dynamic Psychotherapy (ISTDP)

ISTDP is derived from Freud’s original psychoanalytic theory and from AIP. It focuses on the defensive patterns arising from the original attachment ruptures rather than the ruptures themselves. In ISTDP formulations, the rupture in the attachment relationship causes emotional pain in the child and a retaliatory rage towards the parent(s) for causing the pain. However, because the child also loves the parent(s), s/he feels guilt about experiencing rage towards someone he loves. The rage, guilt, grief, and love are all repressed into symptoms and are submerged under behaviors that enable the child to continue a relationship with his/her parent(s). This process eventually becomes a characteristic defensive system (Winnicott, 1965). Whenever the child is in a situation that has the potential for a rupture of attachment, the repressed rage, guilt, love, and pain from the initial attachment rupture are re-activated. Anxiety is experienced to block the feelings from entering conscious awareness, and the defensive system is automatically triggered to keep the feelings repressed and to avoid or alter the emotionally triggering situation (Glowinski, 2011). Over time, this pattern is automatically activated in any situation that has the potential to trigger the repressed feelings about the initial attachment rupture (Amos et al., 2011).

Anxiety associated with the internal emotional conflict and defensive patterns becomes a psychological problem in the person’s life. Anxiety can manifest in one or a combination of four ways. The most adaptive manifestation of anxiety is tension in the striated muscles of the body (Joseph, 2025). Chronic striated muscle anxiety is associated with a number of physical problems, including fibromyalgia, pain, spasm, chronic headache, and hyperventilation (Abbass, 2005, 2008). In a therapeutic context, striated muscle anxiety is an indication that the person has the capacity to consciously experience the repressed feelings from the attachment rupture(s). Other, more problematic manifestations of anxiety include smooth muscle anxiety that is somatized into the gut, leading to gastrointestinal symptoms including nausea, reflux, cramping, and the urge to urinate and/or defecate (Ravari et al., 2024). The striated muscles remain relaxed. Chronic smooth muscle anxiety is associated with hypertension, irritable bowel syndrome, and migraine (Abbass et al., 2008).

Anxiety may also manifest as cognitive perceptual disturbance (CPD) expressed as tunnel vision, blurred vision, ringing or buzzing in the ears (Beeber, 2018). Physically, the person will appear relaxed as anxiety is not being expressed in the striated muscles but will manifest confused thinking and not be “present” in the room. Chronic cognitive perceptual disturbance is associated with neurological complaints, for which no medical cause can be found, such as dizziness and fainting. In some cases, anxiety may manifest as somatization or conversion^1^ (Axelman, 2012).

In a therapeutic context, the experience of smooth muscle anxiety, cognitive perceptual disturbances, or conversion indicates that a psychological restructuring process is required before the person is capable of consciously experiencing the repressed feelings from their attachment rupture(s). In restructuring, the person is gradually exposed to increasing levels of anxiety via graded exposure to their repressed feelings and helped to develop and maintain a striated muscle anxiety response (Abbass & Haghiri, 2025). Eventually, the patient is able to consciously experience the previously repressed feelings without undue anxiety. Patients may experience only one of the four types of anxiety, or they may experience a shift from striated to smooth muscle or CPD as the repressed feelings move closer to conscious awareness.

In response to anxiety, defenses are automatically activated. There are three main groups of defenses (Hoviatdoost et al., 2020). Isolation of affect is the most adaptive defensive system. Patients are aware that they are experiencing a particular emotion, but they do not know how they are physically experiencing it. Instead of the physical experience of the emotion, patients with isolation of affect experience striated muscle anxiety.

The second major defensive system comprises repressive defenses (Davanloo, 1996). Patients with repressive defenses do not recognize that they are experiencing emotions. Instead, feelings are repressed into the body. Repressive defenses are linked to smooth muscle anxiety, where feelings are internalized/somatized into, for example, nausea, irritable bowel syndrome, depression, headache, or conversion.

The third major system is the projective/regressive defensive system. Patients using this cluster of defenses do not perceive that they are experiencing emotions, but rather perceive that another person is experiencing the patient’s feelings. This most commonly occurs with anger, but any feeling can be projected. Typically, these patients manifest weepiness (tears without feelings of grief), temper tantrums, explosive discharge of affect, and confusion. This defensive system is associated with CPD (Flynn, 2019).

Each patient is carefully assessed with respect to their form of anxiety expression and their system of defense (Frederickson et al., 2018). This provides important information to the clinician about how to proceed with therapy. Patients with much earlier and more severe attachment ruptures will be more fragile and will require a longer and slower-paced therapy. This group has life-long patterns of regressive and projective defenses, including temper tantrums, explosive discharges of affect, self-harm, drug and alcohol misuse, dissociation, and projection of their feelings into others (Davanloo, 1995).

The aim of therapy is to help the patient abandon his defenses to allow the previously repressed feelings into conscious awareness. The conscious experience of these repressed feelings triggers memories associated with early attachment ruptures, enabling these previously repressed memories and feelings to be resolved (Davanloo, 1996). Much of the work is achieved through the transference, through which the rageful feelings towards the original attachment figures may first be expressed. Once the patient accesses his rage, he will also experience pain and grief at the loss of the attachment relationship.

ISTDP follows a stepwise process comprising eight phases, as follows:

i. Inquiry and psychodiagnostic evaluation;
ii. Pressure;
iii. Challenge;
iv. Transference resistance;
v. Direct access to the unconscious;
vi. Systematic analysis of the transference;
vii. Dynamic exploration into the unconscious;
viii. Phase of consolidation.

In the intake phase (inquiry), the degree of the patient’s pathology, the severity of the resistances, the defenses used (e.g., isolation of affect, repression, and projection), and the way in which anxiety is discharged—through striated muscle, smooth muscle, or by cognitive or perceptual distortion [the underlying physiological and neurological mechanisms for discharge of anxiety are addressed in the polyvagal theory of Stephen Porges (2007, 2011)]—are clarified. The therapist begins to challenge the patient by applying pressure to mobilize complex transference feelings toward the therapist. These are related to earlier, unsatisfactory primary relationships that have generated unconscious anxiety and defenses against this anxiety. The therapist’s role is to block defenses as soon as they arise to bring the resistances/defenses into the therapeutic relationship. As anxiety rises, so does resistance; the therapist’s role is to point out and clarify the defenses that are recruited to prevent emotional experience and expression. The patient may become anxious that the therapist will hurt them emotionally, like important people in their past (transference resistance). Working through the emotions of grief, anger, and guilt follows. In summary, both AIP and ISTDP, the central core conflict is defined as the unconscious emotional struggle between forbidden or painful feelings, the anxiety that those feelings evoke, and the defenses used to avoid experiencing them directly. Both psychotherapies use Malan’s triangles of conflict and person as navigational tools in the therapeutic process. Both bring feelings into the here and now of the interaction between therapist and patient. ISTDP has a more structured format and greater focus on the body compared with AIP. The therapeutic action is to direct the resistance into the transference, paving the way for the eventual conscious experience of the transference feelings and the exploration of the unconscious. In both forms of therapy,^2^ severe MPA is conceptualized as a symptom of complex psychopathology that has arisen from early attachment ruptures. The feelings associated with those ruptures and the defensive system that has been built to cope with the emotional pain are the foci of therapy. This does not mean that factors directly associated with MPA are ignored. For younger musicians, the therapist must ascertain whether the current circumstances of learning and performing music are sound, for example, practice, preparation for performance, pedagogy, parental support without pressure, and realistic expectations. For the older, professional musician, other factors must be assessed, including performance-related musculoskeletal pain (Kenny et al., 2014), burnout, relationships, or other mental health issues (for a detailed exposition, see Kenny, 2011; Kenny & Ackermann, 2016; Ackermann et al., 2014).

For the severely performance-anxious musician, it is important to take a comprehensive family history and a history of current life circumstances in order to identify areas of difficulty that are affecting the severity of MPA. A detailed intake assessment is presented in the first case vignette. The other two cases demonstrate the process and outcome of therapy. The three participants were volunteers who presented for longer-term psychotherapy for severe music performance anxiety that had seriously affected their ability to perform or to continue performing. All three had undertaken previous forms of therapy with no effect. The hypothesis was that the music performance setting re-triggers unprocessed feelings related to early attachment trauma, and that performance anxiety can be a manifestation of the emergence into consciousness of these powerful early feelings that are triggered by the transference of these feelings into the audience. Working through and the resolution of early attachment trauma, which would in turn permit the development of a more compassionate self-concept, will reduce the MPA without direct therapeutic focus on the MPA symptomatology.

## 2. Case Vignette: Intake Assessment and Formulation

To demonstrate the complexity of individual psychodynamics of musicians suffering from severe MPA, I present a detailed history taken from a pop musician, Callum, 26, who was the lead singer and songwriter in a successful rock band until his voice failed him. Callum presented with voice problems, panic attacks, depression, and a pervasive sense of self-doubt. He described a “streamlined” but emotionally distant family, characterized by achievement, conformity, and a lack of validation for Callum’s chosen path. His father was an ENT (ear, nose, and throat) specialist, and his two older brothers were lawyers. He did not mention his mother. Callum described himself as a “misfit” and “black sheep” within his family and sought therapy after experiencing a severe depressive episode following the sudden death of a close friend/lover while abroad, and his inability to continue singing. Below are selections from Callum’s narrative:

Recently, I’ve started having panic attacks… in the time leading up to a gig, I start to feel very separated and vague; my body is just reacting and I’m not quite there. It’s a really bizarre feeling and difficult to put into words … When I’m under pressure I feel really vague. …Then I get brain fog. When I know I’ve reached this point where I’m severely anxious, I get these cold flushes through my hands… For the past two years I get extremely anxious for a few weeks, … and then it passes… Relating to music, specifically last year, I was in a band that was doing a lot of really good gigs and I was at the center of it. I was writing the music; I was organizing it all. It was a seven-piece band with some really good musicians. I’d been overseas for four years travelling, and I got back at the beginning of last year—and suddenly I was confronted with having to be in some sort of musical framework and structure from week to week being at rehearsals and voice being in good condition and I just went into overload. Writing (songs) and having all the stresses that you would have, trying to lead a normal life, waking up early, being concerned about the amount of sleep I was getting. And on top of it all, my voice just shat itself. It began to freak out. That was the way that my anxiety decided to express itself, through my voice because I was the most acutely aware of it on a day-to-day basis. I entered this spiral about it…So that was all of last year—I had all the checks on more than one occasion. I’ve been to a variety of voice and ENT specialists and had laryngoscopies a bunch of times. My dad, who is an ENT specialist, took me to these doctors. They said, “No, there’s nothing there.” Anyway, so this was just an absolute roller coaster, as you can imagine. I’m trying to front more than one band. I was in three bands at the time as well…On top of it all I was coming back from India as well; I’d been overseas for a few years and I was thinking this was all intertwined and at the root of it all, everybody is telling me there is nothing wrong with my voice. And I’m going, “Well, what do I do here?” Sometimes I find it difficult to talk when I’m anxious, and it’s not like a thinking thing. I actually have trouble getting the words out. Late last year I lost one of my closest friends; she died suddenly, and it was around that time that I entered a really severe depression. There were a lot of heavy things happening and I was feeling an immense pressure on my shoulders about this musical thing. I had this amazing band… everyone in the band was saying to me, “You’ve got talent, everything is great. You’re a great songwriter. You’re a great front man. Everything is great.” But I just was systematically undoing it in my head. I just really lost faith in it all. And then this happened… my friend was travelling on a bus through […] and her body just decided that’s it, and she just died. We’d met in India and travelled together for a couple of years. I’d lived with her in […] and there was a romance… the timing was never right but one day we said we would revisit it. Then she died… that’s the bizarre thing about travel relationships—you develop these intense relationships that nobody else in the world knows about… After she died, I woke up in the morning, found no reason to get out of bed…and …considered suicide … I never actually—I was never there, never thinking to myself, “Okay, I’m going to commit suicide” but …just considering the whole meaning of it all… I never felt like I was actually going to go through with it—it was more realizing that all the things that I loved in my life were coming down around me and if I can’t sing and I can’t write music then what have I got to live for? …I had all these things that were coming up on a personal level; singing, being in a band, boiling over and then my friend died and that toppled me over the edge. All the water overflowed out of the pot. Since then, my band came apart and we unofficially broke up at the end of last year… I place the responsibility solely on myself—I brought my personal issues into the band, and I would turn up to rehearsal and not physically be able to sing, like not be able to get notes out of my mouth… and it became infectious, because nobody wanted to be in that environment, where there was no creativity and there was a really bad vibe around. In the last six months… I’ve done a full circle. I took a solid few months off gigging. I was writing music. I tried to get the band back together once or twice, but it just never happened…There’s a couple of guys in that band who are professional musicians, and that always really intimidated me because they would always put me in a situation where I didn’t feel like I belonged there. I didn’t feel like I was legitimate—I didn’t feel like I had earned my right to be there. I was saying to myself, “Why do these really great musicians want to play with me?” They believed in me, but I just butchered it… I’m not good enough. Why are these guys wanting me… they were getting us really good gigs … but I just didn’t believe in it. I thought, “As if these guys want to play music with me.” I had really no faith in myself.

Callum reported an array of distressing symptoms [feeling “separated and vague;” (dissociation); “my body is just reacting and I’m not quite there” (depersonalization/disembodiment), “brain fog” (cognitive perceptual disruption); “cold flushes through my hands” (motor conversion)], which we may conclude are symptoms of panic attacks/dissociative episodes. He talks of going into “overload” and describes how he tries to cope with feeling overwhelmed and uncontained by “trying to lead a normal life…getting enough sleep.” Callum also reports “…find[ing] it difficult to talk… to get the words out” (somatization) and feeling “immense pressure on my shoulders (striated muscle tension).” In short, he was on an “absolute roller-coaster.”

This symptom complex suggested the presence of extreme anxiety in someone who does not have a reliable, secure base. Callum’s anxiety, generated by repressed emotion, manifested in all four ways described by Abbass et al. (2012): (i) tension in the striated muscles of the body; (ii) smooth muscle anxiety that manifests as somatizing illnesses; (iii) cognitive perceptual disruption (CPD) (Eielsen et al., 2025) that manifests as confusion, blanking out, tunnel vision, blurred vision, ringing or buzzing in the ears, and dizziness and fainting and (iv) motor conversion, which manifests in unexplained weakness or other physiologically inexplicable symptoms in the limbs and psychogenic voice disorders (Axelman, 2012).

The next task of therapy is to develop a case formulation and treatment plan.

### 2.1. Attachment History and Patterns

Callum’s narrative suggests a history of insecure attachment, primarily characterized by avoidant/dismissing attachment strategies. This is evidenced by the following:

Emotional neglect: A father who was busy with work, emotionally unavailable, but caring in a clinical sense, taking his son to see ENT specialists. Mother was not mentioned and did not figure in the narrative. This perceived parenting experience led to a belief that he was a “misfit” in his family, a non-achiever, and this contributed to feelings of worthlessness.

Family emphasis on achievement and conformity: The “streamlined” family structure, with its rigid expectations for success and conformity, likely created a sense of pressure and a lack of acceptance for Callum’s unique identity.

Suppression of emotions: While not explicitly stated, the family’s emphasis on conformity and achievement may have discouraged the expression of vulnerable emotions, such as sadness, fear, vulnerability, or neediness.

Self-reliance and idealization of independence: Callum’s decision at age 15 to leave home, to “make my own way” and reject parental support, suggests a defensive reliance on self-sufficiency to avoid further disappointment in his attachment figures and the intrusive experience of emotional vulnerability, possibly a complex amalgam of rage and attachment longing. These self-sufficiency strivings are frequently seen in people with avoidant/dismissing attachment styles.

Disorganized attachment features: Callum described difficulty with talking and singing, voice production, and inability to “say things correctly” Callum simultaneously wants to reveal himself (perhaps to demonstrate his talent and his “place in the world”) and to keep himself hidden to conceal his felt inadequacy and shame at perceived failure (“As if these guys want to play music with me. I really had no faith in myself”). A feature of disorganized attachment is that the individual experiences both approach and avoidance towards the same goal, often leaving him/her confused and frozen in indecision.

### 2.2. Internal Working Models

Bowlby formulated the concept of internal working models (IWMs), which he understood to be abstractions of interpersonal experience, initially with the primary caregivers (Main et al., 1985). IWMs are cognitive frameworks or templates comprising mental representations of the self, attachment figures, and relationships in general. They act as templates for how individuals expect relationships to function and how they expect to be treated by others. IWMs guide our expectations, emotions, and behaviors. Consistent and responsive caregiving leads to the development of secure IWMs, while inconsistent, neglectful, or abusive caregiving leads to insecure or fragmented IWMs, which form the basis for self-concept, or representations of the self as lovable or unworthy and flawed. IWMs also underpin our representations of others as reliable and available or unreliable and rejecting. When we encounter new relationships, our IWMs will generate expectations about the type of interaction we can expect—one of trust and mutual support or one of betrayal and unavailability. Kenny (2011) has argued elsewhere that IWMs form the basis for the musician’s relationship with their audience.

Based on his attachment history, Callum likely developed the following internal working models (IWMs):

Self: Unworthy of love, attention, or validation. “I’m not good enough.” “Why do these really great musicians want to play with me?”

Others: Unreliable, unavailable, and unable to meet his emotional needs. He cannot believe that he can be supported. “They believed in me, but I just butchered it.”

Relationships: Unsafe, disappointing, and characterized by a need for self-reliance. People are not going to show up for you. Callum’s primary attachment figure in adulthood, his “travel lover” died suddenly, leaving him utterly bereft.

Defense mechanisms

Intellectualization: Engages in “philosophical downward spirals” and over-analyzes his situation to distance himself from his emotions.

Avoidance: Rejects his family’s expectations, choosing an “alternative” lifestyle and isolating himself in his music.

Idealization: Tends to idealize others (e.g., other musicians in the band), devaluing himself in comparison. Perhaps he has an unacknowledged idealization of his conformist, successful family.

Denial: Of vulnerability, of need for relationships, and success in the mainstream world. Demonstrates counter-phobic self-sufficiency by leaving home at 15.

Emotional dysregulation and isolation: All other feelings are subsumed by his intense anxiety.

### 2.3. Affect Regulation

Callum’s affect regulation is significantly impaired. He struggles to tolerate anxiety, which leads to the following:

Panic attacks: Overwhelmed by feelings of separation, vagueness, and “brain fog.”

Voice problems: His anxiety manifests somatically in his voice, suggesting a difficulty expressing his true self and asserting his needs.

Withdrawal and isolation: He withdraws from gigs and isolates himself, seeking to avoid triggering situations.

### 2.4. Transference and Countertransference

Avoidant transference: Callum may initially present as independent, self-sufficient, and emotionally distant, testing the therapist’s willingness to be genuinely interested and available.

Testing the therapist’s understanding: Expresses difficulty putting feelings into words, which will test the therapist’s ability to understand.

Countertransference: The therapist may experience a desire to rescue or “fix” Callum or feel frustrated by his resistance to vulnerability.

### 2.5. Central Dynamic Conflict (CDC)

Wish: To be seen, understood, accepted, and loved for his authentic self, particularly by his family.

Response of other: Emotional unavailability, invalidation, and pressure to conform, leading to feelings of worthlessness, rejection, and a sense of being fundamentally flawed.

Response of self: “Misfit” identity, withdrawal, self-criticism, self-sabotage, and a defensive reliance on self-sufficiency.

### 2.6. Triangle of Conflict

Feeling: Rage, grief, and a longing for connection and validation from his family.

Anxiety: Panic attacks, voice problems, and a pervasive sense of self-doubt.

Defense: Intellectualization, avoidance, self-sabotage, and idealization.

### 2.7. Treatment Goals and Interventions

The primary treatment goals would need to achieve the following:

Address the trauma: Talk about the trauma.

Uncover the feelings: Help him to express his emotional state in the here and now of the transference relationship and then complete the triangle of person, so that his voice will be heard.

Increase capacity for intimacy: Help him relate to others.

Challenge defenses: Help to address the anxiety in the current moment within the transference.

### 2.8. A Therapist, Being Attachment-Informed

⮚ Acknowledges his grief of never feeling loved: This on a background of having lost his one true love.
⮚ Help him understand that his feelings are justified: Validating his need.
⮚ Challenge his defenses: By identifying and labeling the behaviors.
⮚ Integrate thoughts and feelings: This is the phase of meaning-making in therapy. Despite the care he exercised, his “voice just shat itself. It began to freak out. That was the way that my anxiety decided to express itself, through my voice because I was the most acutely aware of it on a day-to-day basis.” Callum’s state of disorganized attachment was embodied in his voice, which was both the nurturer/giver and the tormenter/withholder. Callum’s preoccupation with his voice and his throat could also be seen to represent a bid for his father’s attention. His father responded to Callum’s distress in an “organized insecurity” (typically avoidant) fashion (Holmes, 2010), i.e., Father was to an extent “there”, taking his son to an ENT surgeon, but perhaps in an emotionally distant and somatizing way. He was unable to respond to his son’s emotional distress. The very part of himself that Callum wanted his father to love and validate was also the part that both manifested his vulnerability, and perhaps, too, like the inconsolably crying infant, wanted to attack and debase and baffle his father in protest at his father’s emotional unresponsiveness.

The two vignettes presented below demonstrate how the case formulation becomes the plan for therapy. This formulation for Penelope used a fusion of the principles of attachment-informed psychotherapy and intensive short-term dynamic psychotherapy (ISTDP).

## 3. Case Report 1: Penelope, 21, a Tertiary Level Music Student

### 3.1. Background

Penelope is a 21-year-old Law/BMus student who presented with extreme MPA. She is a classical pianist and singer. She gained her AMusA at 14 years. She now regularly breaks down during performances and feels that a career in the performing arts is looking increasingly bleak. She was only doing a double degree in law to appease her parents.

Penelope is the elder of two daughters—her sister (Elissa) is 19 and studying medicine. This is an immigrant family. Mother is the sole breadwinner, working as an interpreter. Father has bipolar disorder and has not worked at all in 20 years. He is a failed musician who also suffered from extreme MPA; he also started a degree in his early adulthood that he never finished. He sits around smoking and playing his guitar all day when he is not sleeping, which is much of the time. His moods are labile, and he is really a third child in the family. He has problems with alcohol abuse, during which times he becomes verbally abusive, particularly towards Penelope, who provokes him. He lives the life of an invalid, relying on his wife to earn money and run the family.

Penelope’s mother calls him a “lame dog”—she told Penelope that she does not regret marrying him because her situation has made her strong. She asked her mother why she did not leave him, and her reply was that she could not throw a lame dog onto the street.

Penelope has expressed anger towards her father for failing her. She seems somewhat enmeshed with her mother and sister (“we always did everything together”), although recently she has expressed envy and anger towards her sister, who knows her own mind and is pursuing her goal to become a doctor. Penelope wanted to pursue an acting career but knows that this is not realistic. Her family is totally opposed to the idea.

Penelope described her MPA as “demons” on her shoulder, who whispered destructive comments in her ear every time she tried to perform, such as “Why bother? You are going to botch this performance like all the others. You are a failure.” She said that she tries to flick the demons off her shoulder, but they “clawed and clung to her clothes and climbed right back” up onto her shoulder, all the while denigrating her and her incapacity to perform. Penelope then said that the voices were so difficult to silence because she thought that they were speaking the truth—that she really was a failure, useless, and without purpose. “How can you combat the truth?” she asked. I asked her whose faces she saw on the demons on her shoulder. Without hesitation, she said she saw her father’s face and the faces of some of her music teachers who had had such high expectations and who had now expressed such disappointment in her.

Penelope had 10 sessions of cognitive behavior therapy in the previous year and liked the structure and homework, but the effects were short-lived, and she felt devastated by her failure to resolve her issues with this approach. She has expressed suicidal ideation on several occasions. Penelope stated that “music was my main solace in life and without it, I feel bereft.” Below is a description of the formulation and therapy for this young musician.

### 3.2. Assessment of Suitability for ISTDP

Based on the initial assessment, Penelope was considered suitable for ISTDP due to the following factors:

Articulate and motivated: Was able to verbalize her difficulties and express a desire for change.

Psychological insight: Possessed considerable psychological insight into her problems.

Capacity for relationship: Demonstrated some capacity for rapport and therapeutic alliance.

Presence of anxiety: The presence of manifest anxiety indicated that her defenses were being challenged and that she was on the verge of experiencing her core emotions.

Access to the unconscious: Showed early access to the unconscious.

### 3.3. Central Dynamic Conflict (CDC)

Penelope’s central conflict revolves around her rage and resentment towards her parents, particularly her father, stemming from early attachment failures, their emotional unavailability, and their failure to provide adequate support and validation. This rage is heavily defended against due to guilt, fear of rejection, and fear of her own destructive impulses. Penelope offered this appraisal of her relationship with her parents:

I have not been able to separate from my parents… we are overinvolved in each other’s lives… I do not have a separate identity from my parents… my parents are living through me. I don’t have a life of my own. I have no privacy… but my parents would feel very hurt if I moved out.

Penelope worried that she would end up like her father, “a hopeless mental patient who had done nothing with his life”. Penelope’s rage towards her father was close to awareness, but she defensively turned the anger in on herself, deriding herself for being defective and a failure, clearly attributions that belonged to her father. She was also angry that her mother was more interested in her father’s feelings than hers and expected more of her than her father, who was an adult.

### 3.4. Manifestations of Anxiety

Penelope experienced anxiety across a spectrum of manifestations, indicating the depth of her conflict and the strength of her defenses.

Somatization: “Globus” sensation (lump in the throat), striated muscle tension, sighing respirations.

Cognitive/perceptual: Self-denigration, feelings of worthlessness, suicidal ideation, feeling like a “waste of space” and “a useless piece of carbon.”

Affective: Intense rage, frustration, hatred, anxiety, guilt, shame, envy, depression, helplessness.

Behavioral: Breaking eye contact, covering smiles, practiced social poise (tactical defenses), breaking down in musical performances, self-sabotage, binge eating, seeking quick fixes from therapy (strategies/homework), intellectualization.

### 3.5. Defenses

Penelope employs a range of defenses to avoid experiencing her core emotions, particularly rage and grief.

Isolation of affect (Intellectualization): She analyzes her problems with “remarkable intellectual insight” but struggles to feel them.

Turning anger in on herself: Self-denigration, self-criticism, viewing herself as defective and a failure.

Tactical Defenses: “Cover” smiles, practiced social poise, breaking eye contact—used to maintain a socially acceptable facade and avoid genuine connection.

Rationalization: Attempts to find logical explanations for her parents’ behavior (“I can’t be angry with my father; he cannot help himself”) to avoid experiencing the underlying rage.

Detachment (emotional distancing): Breaking eye contact, becoming self-denigrating to create distance between herself and the therapist.

Helplessness: A regressive defense triggered by anxiety, presenting as passive and unable to take action.

Idealization: Initially idealizing her sister Elissa, masking underlying envy and hostility.

Acting out: Penelope described a wish to do a home invasion on her father with masked people who would put him through some torture trials. Perhaps her regular performance breakdowns are a way of acting out her pain and insecurity and projecting her feelings of shame and inadequacy onto her music teacher and parents.

### 3.6. Transference and Capacity for Relationship

Penelope presents with a complex transference pattern, demonstrating both capacity for relationship and significant resistance.

Quick-fix expectations: Impatient for answers, solutions, and homework from the therapist, indicating a wish for a parental figure to solve her problems quickly.

Disappointment and criticism: Expressing disappointment when the therapist does not provide a quick fix, mirroring her disappointment in her parents.

Anxiety in the face of closeness: Breaking eye contact and becoming self-denigrating when painful feelings are stirred in the therapeutic relationship, suggesting a fear of intimacy and vulnerability.

Capacity for rapport: The therapist notes a “significant deepening of rapport” and strengthening of the “unconscious therapeutic alliance,” suggesting Penelope has the capacity for genuine connection and trust.

Testing the therapist: Projecting onto the therapist and then accusing her of not helping to bring experiences of her current life into the transference.

### 3.7. Capacity to Tolerate Anxiety and Regulate Affect

Penelope’s ability to tolerate anxiety and regulate affect is initially limited.

Low tolerance for anxiety threshold: She quickly becomes overwhelmed by anxiety, resorting to defenses and seeking immediate relief. Binge eats to self-soothe.

Difficulty with affect regulation: She struggles to acknowledge and express intense emotions, especially rage and grief.

Improvement over time: As therapy progresses, Penelope demonstrates an increasing capacity to tolerate anxiety and acknowledge her emotions. The reduction of the “lump” in her throat and her ability to perform without panic suggest improved affect regulation.

### 3.8. Triangle of Conflict

Feeling: Rage and resentment towards her parents, particularly her father, and the associated guilt and fear.

Anxiety: Somatic symptoms (globus, muscle tension), self-denigration, suicidal ideation.

Defense: Intellectualization, rationalization, tactical defenses, turning anger inwards.

### 3.9. Triangle of Person

Penelope: Experiencing the conflict, employing defenses, and attempting to manage her emotions.

Father: The primary target of her rage and resentment, embodying emotional unavailability, failure, and dependence.

Mother: Contributing to the conflict through her enmeshment with father, her emotional unavailability to Penelope, and her expectations for Penelope to be emotionally responsible for the family.

Therapist: Feelings towards her parents were enacted in the transference, allowing them to work through in the present.

### 3.10. Interventions

Based on the ISTDP model, the therapist employed the following interventions:

Pressure: Actively challenged Penelope’s defenses (intellectualization, rationalization, tactical defenses) to create a therapeutic impasse and activate anxiety.

Clarification: Helped Penelope identify and label her emotions, particularly rage and grief, and trace them back to their source in her early relationships.

Challenge of defenses: Pointed out Penelope’s defenses as they manifest in the therapeutic relationship, highlighting how they prevent her from experiencing her emotions and connecting with others.

Anxiety regulation: Provided support and containment to help Penelope manage her anxiety and avoid becoming overwhelmed.

Head-on collision with feelings: Guided Penelope to directly experience her feelings, particularly rage towards her parents, in the safety of the therapeutic relationship.

Capacity building: Helped Penelope develop new, adaptive ways of managing her emotions and relating to others. Towards the end of therapy, Penelope stated that she had become a “positive nihilist.” She also developed more realistic goals for her musical career, accepting that she would not be a concert artist, but could spread the joy of music through other means, for her, through music teaching and performances in local music societies.

### 3.11. Summation

Therapy focused on clarifying the system of defenses Penelope used to defend against her hostile, angry feelings and the self-destructive consequences of such a system, how these characteristic patterns of interpersonal interaction were evident in the therapeutic dyad (i.e., transference), e.g., her disappointment in the therapist at the absence of a quick fix was barely concealed—she would increase her self-critical comments when these negative transferential feelings threatened to erupt.

In response to this focus on attending to her feelings, the defenses she used, and what the defenses were helping her to avoid, Penelope disclosed an urge to self-harm and wished that she were dead. The therapy then focused attention on these feelings and encouragement to take action against helplessness^3^ by pointing out her detachment^4^ in the transference (e.g., Penelope broke eye contact when feeling anxious, vulnerable, or suicidal, and laughed when connecting with painful feelings) and her use of the defenses of rationalization (e.g., “I can’t be angry with my father; he can’t help himself”) and isolation of affect (e.g., “I have a job to do; I should just get up and do it”) as strategies to avoid confronting her painful feelings towards her parents. The therapist gradually increased the focus on the central issues, continually pointing out the patient’s defenses, countering the patient’s rationalizations^5^, and blocking irrelevant and distracting talk.

The challenge for Penelope was to identify her hostility towards her father, rather than avoiding, then rationalizing it and turning the hostile feelings onto herself. Penelope expressed her fear that she would end up like her father—“a lame dog,” passive, and a failure. I made the link between her negative feelings towards me for not giving her a quick fix and the increase in her self-denigration when she became aware of hostile feelings towards me. I then made the T/P link with her father. The triangles of time and person were worked through many times with her father, mother, sister, and me. The therapeutic work with Penelope centered on helping her to access her core emotions, dismantle her defenses, and develop healthier patterns of relating to herself and others. Working with the transference was essential, challenging defenses, and supporting her capacity to tolerate anxiety and express her feelings in a safe and contained therapeutic environment.

At the end of therapy, Penelope had decided to discontinue her law degree and focus on her BMus, after which she would do her Master of Teaching degree to become a high school music teacher. She felt settled in that decision. In her last session, we had the following exchange:

P: So what happened to make me feel so much better?
DK: What do you think happened?
P: It wasn’t just luck, was it?
DK: It has nothing at all to do with luck. It is about motivation and perseverance and commitment and courage and willingness to stare these painful experiences in the face and deal with them.
P: Does it have a name?
DK: Well, if you want a technical term for it, “The penny dropped.”

We laughed and the session ended with Penelope (Penny) clapping her hands, feeling buoyant and saying that she was confident that she could move into the future with hope.

## 4. Case Study 2: Kurt, 55, Professional Violinist

### 4.1. Background

Kurt, 55, had been a member of the premier state orchestra for 32 years and had reached the position of assistant principal in the first violin section. Due to the recurrent ill health of the principal, he had been called upon on many occasions to fulfill this role. He distinguished himself in the position, despite experiencing long-term severe MPA, which he had managed with a range of therapies and self-help strategies, such as daily affirmations, visualizations, and meditation. He was expected to win the position when it was finally vacated by the incumbent. However, on the day of the audition, he “fell apart”, which, to most highly skilled, professional musicians, means that he did not play at his best. There was no performance catastrophe or breakdown. Nonetheless, he failed to win the position. Kurt gave a bleak history of his childhood.

When I was a kid, I was bred that kids should be seen and not heard. You’re not allowed to cry or have your own thoughts. You just go outside and shut up. I don’t want to hear about you and all that sort of shit… I had a really cruel childhood… I was just abused as a kid, both physically and mentally; I was abused and it hurt. I never got listened to; never got understood. My father beat me, and my mother was just crazy… she was fucking crazy, mad, in and out of the loonie bin.

Kurt identified the source of his emotional pain in his early depriving and abusive relationship with his parents. There was no empathy or attunement, only relentless criticism and the expectation that he would “make himself as small as possible, so as not to be nuisance.” Imagine the conflict in a performing artist who has been “bred” to be seen and not heard and abused for expressing himself!

### 4.2. Central Dynamic Conflict (CDC)

Kurt’s central conflict stems from early childhood abuse and neglect at the hands of his parents, who enforced a “seen and not heard” rule and actively punished his self-expression. This resulted in deep-seated rage and grief related to feeling unloved, unheard, and fundamentally worthless. The core wish is to be seen, heard, understood, and loved.

### 4.3. Manifestations of Anxiety

Kurt experiences anxiety in a variety of ways, demonstrating the pervasiveness of his conflict.

Striated muscle tension: Evident in the therapy (the therapist consistently notices this).

High blood pressure/hypertension: This is noted at the beginning of therapy, but the somatic tension cannot be ignored.

Cognitive perceptual disturbance: Blurred vision, brain does not recognize the notes, separation of cognitive and motor functions, e.g., “I did an audition a while ago and I lost the normal feeling in my arm. I couldn’t explain it. It was like it was somebody else’s arm—I couldn’t control it;” e.g., 2 “Even if I know a piece or a part really well, it’s almost like my brain sees it on the paper and doesn’t recognize it. The message doesn’t get through.”

Emotional: Anxiety, “that fear feeling,” slight hostility, sadness, hopelessness, powerlessness, feeling exposed, feeling judged.

Behavioral: Trying to sit still and shut up in anxiety-provoking situations, withdrawing from people, rationalizing failures, turning anger in on himself, practicing self-affirmations and visualizations. In an audition that required small motor skills, his bowing forearm did not work, causing a loss of sensitivity and resulting in choking.

### 4.4. Defenses

Kurt relies on several defenses to avoid experiencing his core emotions of rage and grief related to his early abuse:

Passivity/helplessness: “I just sit still and shut up,” presenting as if he has no control over his anxiety or his life.

Rationalization: Justifying his anger and making excuses for the judges. “I have sat in their chairs.”

Turning anger in on himself: “I guess I feel it’s my fault.”

Detachment/emotional distancing: Describing feeling “separate” from parts of his body, becoming emotionally unavailable in relationships.

Idealization/intellectualization: “So you’re not really a friendly guy.” This is Kurt becoming intellectual and starting to idealize the therapist to try to get his needs met.

### 4.5. Transference and Capacity for Relationship

Kurt initially presented with a guarded and resistant transference but began to thaw as the therapist persisted.

Testing the therapist: Implying that the therapist was not “friendly” during a session.

Emotional distancing: Building emotional walls with people to reduce vulnerability.

Unconscious therapeutic alliance with the therapist: Wanting to get better but fighting against his adaptive impulses.

### 4.6. Capacity to Tolerate Anxiety and Regulate Affect

Kurt had limited capacity to tolerate anxiety and regulate affect and became anxious quickly. He was unable to tolerate physical feelings, but as the therapy progresses, he demonstrates improved affect tolerance.

Low anxiety tolerance: Easily overwhelmed by anxiety.

Limited affect regulation: Difficulty acknowledging and expressing emotions, especially rage and grief.

Improved over time: Starts to acknowledge that things are bad.

### 4.7. Suitability for ISTDP

Based on the trial therapy transcript, Kurt was assessed as moderately to highly resistant, but a suitable candidate for ISTDP.

Somatized affects: Expressing his anxiety is an issue, evidence of CPD and somatization.

Intellectualization: He is a musician who intellectualizes what is going on in him and who has searched for cognitive solutions for his MPA (self-affirmations, increasing practice).

High level of resistance: Does not yield to the therapeutic process easily even though highly motivated.

### 4.8. Triangle of Conflict

Feeling: Rage and grief related to early childhood abuse and neglect.

Anxiety: High blood pressure, somatization, muscle tension.

Defense: Powerlessness, rationalizations, turning anger inward, detachment.

### 4.9. Triangle of Person

Kurt: Experiencing the conflict, defending against his feelings, and struggling to connect with others.

Mother and Father: Enforcing a “seen and not heard” rule, abusing him verbally and physically, and leading him to see any form of self-expression as dangerous.

### 4.10. Therapeutic Interventions

Clarification: Helped Kurt identify and label feelings.

Pressure: Challenged his defenses, causing him to experience an increased level of anxiety.

Affirmation/Support: Support his attempts to engage with trauma.

Head-on collision: Relentlessly challenge defenses that maintain self-sabotage.

This formulation highlights Kurt’s early experiences of abuse and neglect and how these experiences continued to affect his current life. The therapist’s goal was to help Kurt become aware of his defenses, experience his core emotions, and develop healthier patterns of relating to himself and others. Towards the end of his therapy, Kurt had a major solo performance. He described his experience to the therapist in his last session:

I remember you said that the anxiety feeds off a certain guilt that I have, which I have recognized… as trying to kill off my mother because of feelings of abandonment. I can clearly see that because … whenever I had a performance, I would try to deal with issues that were closest to me, like the physical things and doing meditation, but there was always this underlying feeling that there was something else that I could not deal with and that overrode all the other techniques that I had. So, it feels like I’ve got to the root of the problem. It goes way, way back to early on and that is why it is inexplicable to the intellectual mind. I could never rationalize it or deal with it intellectually. It has always been there, a backdrop that I couldn’t get rid of, so in those ways I felt a certain palpable thing yesterday. I was really pleased with my performance and everybody said, “Fuck, you played well.” And I thought, “Jesus, what’s happened?” I played the best yesterday that I have ever played in public, ever … ever.

In his description of his performance experience, Kurt demonstrated profound insight into his emotional process and the impediments from his early attachment experiences that were holding him back from his best performance. Kurt’s early attachment relationships were dominated by neglect and abuse from those he loved. This caused him emotional pain and feelings of murderous rage towards his parents. However, because he also loved his parents, he experienced unconscious guilt about his rage. Initially, these feelings caused anxiety; gradually, he developed patterns of avoidance, passivity, helplessness, and compliance in order to reduce his anxiety, protect his parents from his murderous feelings, and punish himself for the guilt he felt about having those murderous feelings towards people he loved. As he grew up, these defenses became solidified into his characterand, to a degree, allowed him to function in his relationships, but denied him the full experience and expression of his loving feelings in his most important relationships. However, on stage in front of an audience, a situation that is all about judgment, Kurt’s defenses were useless. As a professional musician, he could not become avoidant, passive, helpless, or compliant and perform at the level required. He was, therefore, left defenseless in a situation that stirred up earlier feelings of rage and guilt about the negative judgments he experienced from his parents as a child. Without the defenses to repress these feelings, unconscious anxiety was his only mechanism to keep these feelings repressed. Kurt therefore experienced anxiety whenever he had to perform. In his relationship with the therapist, Kurt’s unconscious feelings were mobilized as the therapist tried to reach towards an emotionally close relationship. This activated Kurt’s defensive template, allowing the therapist to help Kurt identify and examine his anxiety and defenses and how they were creating an emotional barrier between Kurt and the therapist. Kurt was encouraged to overcome his defenses and to fully experience them with the therapist. As the defenses were overcome and the unconscious feelings were consciously experienced, his early memories became accessible, thereby allowing Kurt and his therapist to work through previously repressed memories and fantasies from his early life. As this occurred, the anxiety and defenses that previously kept this material repressed became redundant and were relinquished, leaving a more integrated and less anxious and defended person. Kurt experienced the benefits of these changes immediately in his first solo performance after his therapy.

## 5. Conclusions

A person with unconscious, unprocessed emotions from early life does not distinguish the past from the present (Kohut, 2018). Patients interact with people in their lives in the present through virtually the same emotional and defensive templates that they developed as children. When they commence therapy, those templates quickly assert themselves in the transference (i.e., relationship between therapist and patient) (Wallin, 2007). The therapeutic benefit of mobilizing complex transference feelings is the ability to directly examine the unconscious and the unconscious therapeutic alliance in the present. These case studies provide support that moderate to severe performance anxiety, in at least some cases, has its origins in unresolved complex emotions and defenses arising from ruptures to early attachment relationships and that attachment-informed psychotherapies are suitable interventions for some of these musicians. A limitation of the study is that longer-term follow-up after conclusion of the therapy was not possible; it reports a small case series, and the therapy is relatively labor-intensive and requires a skilled and experienced therapist. Nonetheless, there is a great deal to be learned from detailed case reports of psychotherapy outcomes that may be incorporated into shorter, less intensive programs. Although suitability for these forms of therapy is assessed at intake, further research is required to identify those more likely to benefit from these forms of therapy.

## Data Availability

Data comprised transcripts of interviews and therapy. For ethical reasons and confidentiality, the recordings and transcripts cannot be made available. Names and minor details have been changed to preserve privacy.

## Notes

1 Somatization refers to the process through which psychological distress and emotional conflict manifest as physical symptoms affecting multiple body systems (gastrointestinal, cardiovascular, respiratory, pain). These symptoms occur because emotions and anxiety are discharged into the body (via autonomic or smooth muscle pathways), resulting in chronic pain, headaches, irritable bowel, or other medically unexplained symptoms. Conversion involves the unconscious transformation of intense emotional states—often forbidden feelings like rage, guilt, or grief—into physical symptoms that cannot be explained medically. Typical conversion symptoms include sensory loss (e.g., blurred vision, numbness), movement problems (called motor conversion), such as non-epileptic seizures, loss of speech, or paralysis, none of which have an identifiable neurological cause.

2 The formulations and therapies described in this paper can only be conducted by a skilled therapist trained in psychodynamic metapsychology and AIP or ISTDP.

3 Helplessness is a regressive defense triggered by excessive anxiety.

4 Detachment refers to emotional distancing as a defense against intimacy.

5 Rationalization is typically a tactical defense that finds intellectual explanations for problems devoid of affect.
